# Mental Health Amidst COVID-19: A Review Article

**DOI:** 10.7759/cureus.33030

**Published:** 2022-12-28

**Authors:** Shraddha Patil, Preeti Thute

**Affiliations:** 1 Anatomy, Jawaharlal Nehru Medical Collage, Datta Meghe Institute of Medical Sciences, Wardha, IND

**Keywords:** internal migrant, psychological disorders, contagious disease, frontline workers, mental health

## Abstract

Outbreaks of infectious diseases confined to a particular locality are not unusual. Respiratory infections such as tuberculosis or community-acquired pneumonia are known in developing and underdeveloped countries. However, COVID-19 infection had globally created havoc due to its high rate of transmission and serious consequences on physical and mental health paralyzing the healthcare facilities of not only developing but also developed nations. This created a sense of uncertainty and insecurity in the public globally, adversely affecting the mental health of almost every individual. It is genuinely obtrusive that the COVID-19 pandemic brought about a global lockdown, adversely affecting the psychological health of the public. Some pandemic-related stressors affect nearly everyone. This review aims to study the effect of the COVID-19 pandemic in terms of psychological well-being and its overall effect on society, thereby making it essential to lend them a helping hand.

## Introduction and background

The first case of coronavirus infection was reported in December 2019 in Wuhan Province in China, which escalated in a short span globally, earning the title of a pandemic affecting all segments of society. As the incubation period of the virus and the rate of its transmission among human beings were completely unknown, concerns and anxiety grew worldwide. Unprecedented large-scale quarantine measures in all predominant towns had restricted human beings to their houses to check its transmission, inflicting a bad mental impact [1]. Many studies were conducted on the psychological status of the people in the course of situations, including isolation, lockdown, and quarantine. A study published in *The Lancet* in 2020 concluded that isolation from cherished ones, lack of freedom, lack of interest, and unpredictability worsened a person’s psychological status and had long-term detrimental consequences such as psychosis and suicidal tendency in adults. However, youngsters who were separated from their guardians, such as those infected with or suspected of being contracted COVID-19, and who were isolated in nearby hospitals or observation centers and children whose caregivers contracted COVID-19 or had passed away and were, therefore, under the care of charitable groups or NGOs were at greater risk of mental health problems [2].

## Review

Effect of misinformation

Unique *info-demic* misinformation on social media spreads swiftly and makes a solution tougher to achieve, posing a primary hazard to public psychological health in the course of this disaster [3]. Users of social media may be less prone to verify the information before sharing it if they are motivated by self-promotion or fun. This act can lead to a lack of trust in healthcare individuals, healthcare mandates, and health officials [4].

Effect on internal migrant workers

The COVID-19 pandemic posed the already susceptible migrant population with great challenges [5]. Many jobless, unqualified, and illiterate populations migrate temporarily from villages to metro cities. During the pandemic, mandatory lockdowns and restrictions on traveling to combat the spread of the virus resulted in stigma, isolation, loss of income, and risk of psychological health issues, including suicide [6,7]. Many biological, social, and environmental factors had an impact on psychological health issues, and these were intensified during the pandemic for migratory workers [8].

Effect on jobless people

Millions of people became jobless because of the pandemic [9]. A drop of 24.7 million jobs and a growth of 5.3 million jobs were reported to be the worst- and best-case situations, respectively, at the press launch of the International Labor Organization (ILO), on March 18, 2020. In the worst-case situation, unemployment could grow up to 5.88% at the growth side of about 2,135 suicides [10]. Moreover, during COVID-19, job sectors were affected differently due to economical and productive pandemic consequences. Experts pointed out that those who had pre-existing psychological illnesses and others who never had any issues related to psychological health would be more likely to develop COVID-19 [11]. In their study, Gunnel et al. accurately predicted how the entire populace was affected by psychological health outcomes during a pandemic. Therefore, data from the research for innovative evidence-based tactics were important to minimize the ill effects on mental health due to the pandemic [12].

Effect on healthcare workers

As already recognized, COVID-19 is a contagious disease. Elements associated with the risk of contagion within the administrative centers and implementation of preventive measures purpose limitless psychological fitness concerns, for example, the shortage of personal protective equipment (PPE) kits, the bodily load prompted through carrying those kits, confined remedial options, concerns of being infected and the possibility of this unfolding in own circle of relatives, arguments among protection strategies and choice to offer guidance, pressing multitasking, long duty hours, and social taboo of frontline workers in these hazardous circumstances can deeply harm their psychological health. In the latest instances, doctors, nurses, and different frontline workers have been pressurized by the general public. Many frontline workers who were staying in rented houses were compelled to leave their houses. Staying far from their homes and serving people in such a bad state of mind would possibly, without a doubt, have an impact on their psychological health, resulting in several behavioral (e.g., influences outcome), bodily (e.g., gastrointestinal disturbances and headaches), and mental reactions (e.g., frustration and depressed temper) [13]. Moreover, many studies reported that the most important worrying point related to COVID-19 was the stigma attached to the progressive increase and alarming spread [14,15]. As a result, there may be an extended threat of mental misery, emotional exhaustion, depressive signs and symptoms, and anxiety [16-18]. Many individuals experienced discrimination in the workplace due to this COVID-19-related stigma, which was found to affect their self-efficacy and, in turn, income, as supported socially [19].

Lu et al. [20] conducted a study on 2,299 respondents, which included the evaluation of the tiers of fear, tension, and despair of medical examiners with the ones of managerial and administrative employees. Results showed significant variations. Frontline workers posted in high-risk units (with direct and extended contact with COVID-19 patients) were found to be affected by more from fear (*P* = 0.024), anxiety (*P* = 0.005), and depression (*P* = 0.007) compared to nonclinical health workers and also were more anxious (*P* = 0.026) than the medical healthcare workers with low risks [20]. Suicidal cases were found to increase rapidly due to the COVID-19 infection fear, financial constraints, and pre- and post-lockdown work-associated pressure. Therefore, this pressure should in no manner be underestimated [21]. A survey by Tan et al. confirmed that 95% of the respondent patterns changed into much less pressured and stricken if returning to a sanitized, ventilated, and prevention-aware administrative center [22]. Huang et al. concluded that an enterprise that can pay interest toward its workers' fitness might achieve greater profits, but if workers' fitness is not taken into consideration, then it may encounter loss in its business [23]. In their study, Sasaki et al. [24] observed that extensive preventive measures for COVID-19 infection in the workplace lessened related anxiety with improved work performance and, in turn, help to promote and maintain the psychological fitness of the workers [24].

Effect on women

Wilson et al. [25] studied the prevalence of psychological illnesses in healthcare workers during the COVID-19 pandemic and observed that the risk of stress, anxiety, and depression was more prone to women healthcare workers and women staying in a hostel facility [25]. Women who were in the postnatal period, pregnant, or affected by miscarriage or were going through intimate partner violence (IPV) could also be at risk of mental health problems [26].

Pregnancy

The perinatal period (pregnancy and the first year after delivery) is a time when women are most vulnerable to mental health issues. An estimated one in every seven perinatal women is affected by depression, anxiety, and distress. Women who are pregnant with a high-risk pregnancy are at an even higher risk of depression. Due to the lack of definitive data on the effects of COVID-19 during pregnancy, the COVID-19 pandemic causes heightened dread and a diminished sense of control for many pregnant women [27].

Postpartum

Postpartum depression is protected by social support. The level of social support has a strong and inverse relationship with the severity of postpartum depression symptoms [28]. When dealing with severe hormonal changes, sleep loss, and changes in the family dynamic and role allocation, effective postpartum social support might involve relying on family, friends, or paid professional help to receive some reprieve from extra obligations. This is no longer an option for many females, who are suddenly juggling several roles with minimal aid due to pandemic-related, stay-at-home or shelter-in-place directives [1].

Miscarriage

Women who miscarry have higher rates of anxiety, depression, and post-traumatic stress disorder (PTSD) than women who retain healthy pregnancies [29]. Women with nonviable pregnancies can choose between expectant care (natural miscarriage), medicinal therapy, or surgical treatment in nonpandemic situations. During the pandemic, more women may choose to miscarry at home to avoid COVID-19 exposure in a medical setting. Natural miscarriage takes a long time than surgical therapy, is more likely to be incomplete, and requires unanticipated surgical intervention and/or transfusions [30]. Women who would have otherwise chosen surgical interventions may now undergo many days of bleeding and cramps, occasionally seeing fetal bits in the material they pass, and often enduring this in more solitude, with less social support accessible. This points to a larger likelihood of mental health issues in the future [26].

IPV

While the world is encouraged to stay at home for protection, for many people, being at home is the least safe alternative. These are victims of intimate relationship abuse, most of them are women, who are now more exposed to perpetrators of violence amid a period of unparalleled psychological and economic hardship, as well as having fewer safe havens. In other circumstances, violence may emerge in homes where it had previously been absent [26].

Effect on children

As compared to grown-ups, the impact of the pandemic and lockdown on social and emotional development was observed more in young children and adolescents. Irritation, inattention, and clinginess were observed to be increased in children regardless of their age, while older children were more curious about COVID-19 and hence frequently questioned about the COVID-19 pandemic and its consequences [31]. Jiao et al. [32] conducted a study in which parents' feedback revealed uncertainty, fearfulness, and isolated condition of children during that period. Parents also observed children experiencing sleep disturbances, nightmares, loss of appetite, distress, distraction, and uneasiness due to separation [32]. In a study by Sprang and Silman [33], it was observed that youngsters who had been isolated or quarantined in the course of the epidemic were at a greater probability to be affected by acute pressure disorder (mental shock), adjustment disorder (situational despair), and affliction. Thirty percent of the isolated or quarantined youngsters met the scientific standards for PTSD [33].

Singh et al. [34] studied the psychological effects of the pandemic on children and adolescents. As reported in the literature, the prevalence of COVID-19 infection is less in children than in adults. However, the stress confronting them makes them highly susceptible. The results of many cross-sectional studies showed that the effect depends on many factors such as maturity, level of literacy, known psychological illness, poverty, or being in isolation due to infection or fear of infection. Closure of educational institutes and activity or entertainment centers for long period as preventive measures lead to a negative effect on the quality of education and psychological and developmental progress in children as they experience loneliness, anxiety, and unpredictability. Due to online education, the use of online games and social media predisposes them to be at greater risk of psychological illnesses. Children with pre-existing psychological illnesses cannot cope with variation in the environment, leading to its exacerbation. Children who are quarantined are at a high risk of psychological challenges [34].

**Figure 1:**
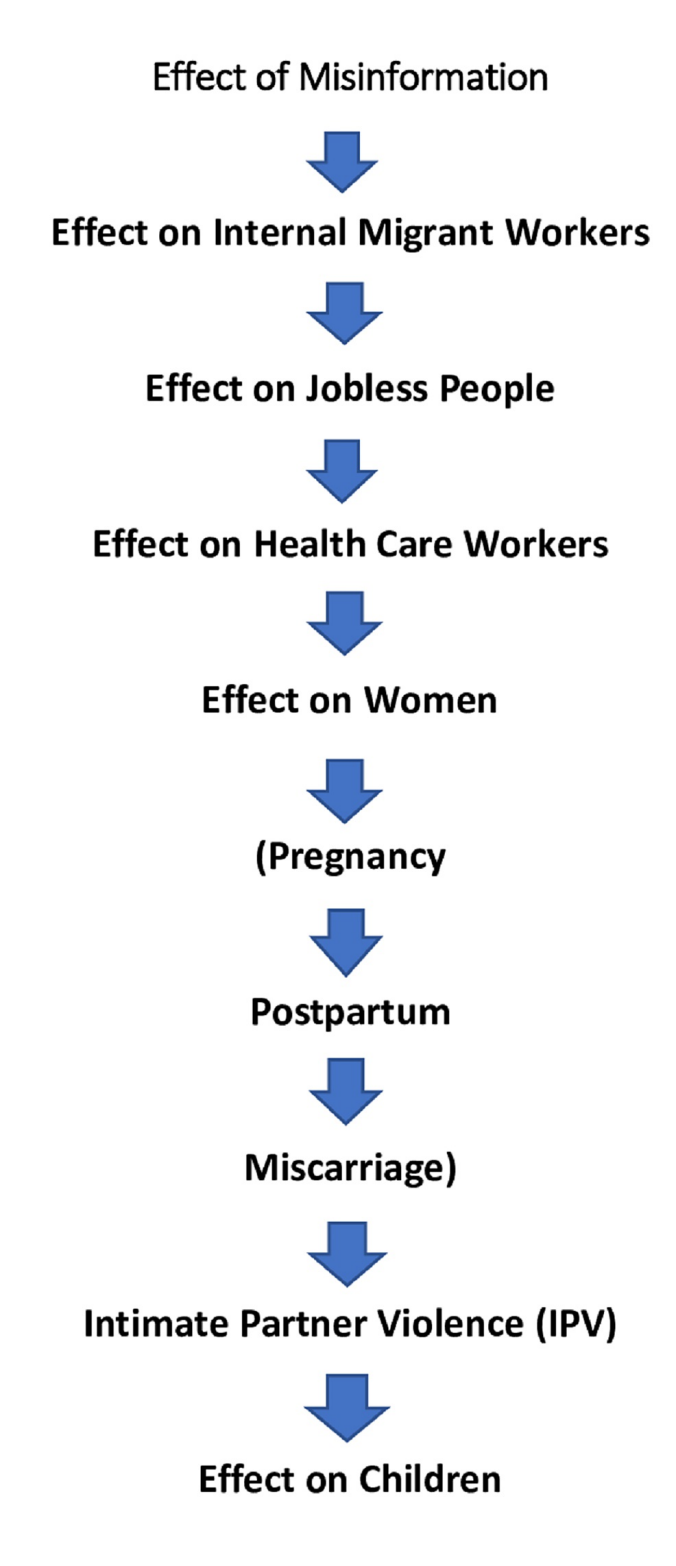
Triggers of mental health. Figure credits: S. Patil and P. Thute

Fox et al. [35] researched to determine the frequency of despair, tension, and pressure and tried to triumph over them. The study determined that 25.1%, 28%, and 11% of the individuals have been reasonably to extraordinarily depressed, anxious, and pressured, respectively. Consumption of too much alcohol in a brief time frame is significantly and undoubtedly related to depression, anxiety, and pressure. Currently, hired repute is also associated with depression and anxiety. Hundreds of heaps of jobs will be misplaced once COVID-19 is repealed [35]. Das [36] put forth a theory in which he stated that salaries will be cut and that personnel raises will be frozen. Unpredictability and fear of future life could have resulted in more depression and anxiety. Employees also are not able to satisfy the time limit/goals, and work stress is likely to have contributed to their depression and anxiety. The finding from the National Mental Health Survey of India, 2016, pronounced that females are more prone to be afflicted than males [37]. The most important reason behind this is that generally females deal with the family chores (including child care, laundry, cleaning, and cooking) as well as their professional fronts due to typical sociobehavioral standards that are predominating in most Indian communities. Males in Indian culture minimally take part in family chores. Now, talking about the positive aspects, the deliberate slowing down of each day's routine may be useful. For example, holidays and weekends are set to limit pressure and tension. Likewise, a few spiritual and religious traditions inspire mindfulness, simplicity, and solitude, to grow well-being [37]. It is consequently plausible that for a few human beings, the lockdown ought to provide a brief/everlasting remedy from each day's hassles and pressure or even result in growth in well-being. It is, therefore, equally vital to find out shielding elements that can limit the unpleasant outcomes closer to the bad influences of the lockdown (Figure 1 ).

## Conclusions

This review revealed that the magnitude of the mental illness caused by the COVID-19 pandemic is far more devastating than it appears on the surface. It affects children, youngsters, and adults. Women are being more frequently and adversely affected by the pandemic. It affects all strata of society. During this difficult time of the COVID-19 pandemic, the top priority of authorities should be to address the needs of mental health. It is already making excellent progress in containing the spread of the COVID-19 pandemic. Appropriate measures should also be taken to promote the psychological health of the populace. In the setting of online mode, psychiatric services to be made available by psychological health experts may be taken into consideration, keeping in mind the primary additives to triumph over psychological health-associated troubles. The importance of social support must be made clear to the general public, and viable mechanisms that can contribute to social support must be catered to the extent possible. It is beneficial to counteract the psychological signs and symptoms with the use of supportive therapy, reassurance, accurate data, and treatments for those in despair or tension. Providing correct statistics is most important to limit the experience of uncertainty and lack of confidence and increase life satisfaction. For the control of psychological illnesses, motivational interviewing (MI), cognitive behavioral therapy (CBT), and disaster intervention were considered beneficial intervention approaches. Maintaining the conversation with family members is of utmost importance during isolation, and if it isn't possible, then healthcare experts should make an effort to communicate and provide a sense of support. Worldwide, healthcare interventions should be implemented to combat-related psychosocial stress factors mainly associated with isolation or quarantine, fear of infection, and susceptibility to infection among the general public. As media reviews may be emotionally disturbing, misinformation from media or social networking sites should be controlled and monitored. Globally, community-supportive psychosocial measures should be promoted.
